# Low birthweight and preterm birth in young people with special educational needs: a magnetic resonance imaging analysis

**DOI:** 10.1186/1741-7015-6-1

**Published:** 2008-01-30

**Authors:** Michael D Spencer, T William J Moorhead, Rod J Gibson, Andrew M McIntosh, Jessika ED Sussmann, David GC Owens, Stephen M Lawrie, Eve C Johnstone

**Affiliations:** 1 Division of Psychiatry, University of Edinburgh, Kennedy Tower, Royal Edinburgh Hospital, Morningside Park, Edinburgh EH10 5HF, UK; 2 Division of Clinical Neurosciences, University of Edinburgh, Western General Hospital, Crewe Road, Edinburgh EH4 2XU, UK

## Abstract

**Background:**

Although neuroanatomical and cognitive sequelae of low birthweight and preterm birth have been investigated, little is understood as to the likely prevalence of a history of low birthweight or preterm birth, or neuroanatomical correlates of such a history, within the special educational needs population. Our aim was to address these issues in a sample of young people receiving additional learning support.

**Methods:**

One hundred and thirty-seven participants aged 13–22 years, receiving additional learning support, were recruited via their schools or colleges and underwent structural magnetic resonance imaging (MRI). Obstetric records, available in 98 cases, included birthweight and gestational data in 90 and 95 cases, respectively. Both qualitative and quantitative voxel-based analyses of MRI data were conducted.

**Results:**

A history of low birthweight and preterm birth was present in 13.3% and 13.7% of cases, respectively. Low birthweight and preterm birth were associated with specific qualitative anomalies, including enlargement of subarachnoid cisterns and thinning of the corpus callosum. Low birthweight was associated with reduced grey matter density (GMD) in the superior temporal gyrus (STG) bilaterally, left inferior temporal gyrus and left insula. Prematurity of birth was associated with reduced GMD in the STG bilaterally, right inferior frontal gyrus and left cerebellar hemisphere. Comparison of subjects with no history of low birthweight or preterm birth with a previously defined control sample of cognitively unimpaired adolescents (*n *= 72) demonstrated significantly greater scores for several anomalies, including thinning of the corpus callosum, loss of white matter and abnormalities of shape of the lateral ventricles.

**Conclusion:**

Although a two-fold increased prevalence of a history of low birthweight and preterm birth exists within the special educational needs population, other aetiological factors must be considered for the overwhelming majority of cases. Neuroanatomical findings within this sample include qualitative anomalies of brain structure and grey matter deficits within temporal lobe structures and the cerebellum that persist into adolescence. These findings suggest a neurodevelopmental mechanism for the cognitive difficulties associated with these obstetric risk factors.

## Background

Intellectual disability is a lifelong disability that is associated with considerable cost to society and often considerable limitations to the quality of life of affected individuals. A widely employed definition of intellectual disability is that of the American Association on Mental Retardation (AAMR), which requires three criteria to be met: (1) the presence of sub-average intellectual functioning, comprising an intelligence quotient (IQ) of less than 70; (2) the presence of impaired adaptive functioning, comprising deficits within domains such as academic or occupational functioning, social skills and activities of daily living; and (3) an onset prior to the age of 18 years [1]. The prevalence of intellectual disability, as defined by an IQ of below 70, is primarily determined by the statistical distribution of IQ within the population, and a review of epidemiological studies indicated a prevalence of about 3% in school age children [2]. In Scotland, 2.2% of children aged 5–16 years are registered with the Support Needs System (SNS) as having additional support needs (expressed as a percentage of the total child population of the NHS Board areas covered by the SNS) [3].

Low birthweight (less than 2500 g) and preterm birth (less than 37 weeks' gestation) are relatively common within developed countries, affecting approximately 7% (see [4]) and 6% (see [5]) of all births, respectively, and are associated with an increased risk of significant neuropsychiatric morbidity, particularly in terms of cognitive impairments and an enhanced risk of neurodevelopmental disorders such as attention-deficit hyperactivity disorder [6-9].

Low birthweight and preterm birth are also known to be associated with a range of qualitative and quantitative abnormalities of brain structure. Low birthweight is associated with ventricular dilatation, white matter loss and thinning of the corpus callosum [10,11] and cortical thinning in parietal, temporal and occipital lobes and cortical thickening in frontal and occipital lobes [12]. Preterm birth is associated with similar qualitative anomalies, including ventricular dilatation, white matter loss and corpus callosal thinning [13], as well as periventricular leukomalacia and basal ganglia haemorrhage [14], and quantitative abnormalities in terms of reduced hippocampal and caudate volumes [15-17], reduced cerebellar volumes [18,19], reduced cortical grey matter volumes in parieto-occipital regions [20], thinning of the corpus callosum [21], increased temporal lobe gyrification [22] and enlargement of the occipital and temporal horns and body of the lateral ventricles [20,23].

Our understanding of the neuroanatomical and cognitive sequelae of these conditions is largely derived from longitudinal cohort studies, investigating brain structure and cognitive impairments within groups of subjects identified, either at the time of birth or during subsequent recruitment from clinical samples, as being born preterm and/or with a low birthweight. To the best of our knowledge, no study has as yet examined these issues within a cohort of cognitively impaired individuals, recruited as such via the educational system. Consequently, little is understood as to the likely prevalence of a history of low birthweight or preterm birth, or neuroanatomical correlates of such a history, within the special educational needs population.

This study addresses these issues within a clinically well cohort of young people receiving additional learning support at school, recruited as part of a longitudinal study investigating the prevalence and evolution of psychopathology within adolescents with special educational needs because of an apparent intellectual disability in comparison to a control group of normally developing young people [24]. Structural magnetic resonance imaging (MRI) findings reported within this cohort include an increased range and degree of qualitative anomalies of brain structure in adolescents receiving educational support as compared with the controls [25], structural abnormalities within right cerebellar, left parieto-temporal and posterior corpus callosal regions in intellectually impaired subjects as compared with controls, and abnormalities within the thalamus and left superior temporal gyrus of intellectually impaired subjects with (as compared with those without) autistic features [26], and associations between symptom severity of anxiety, incoherence of speech, hallucinations and delusions and grey matter density in a range of brain regions, including lateral and medial temporal lobe structures and the thalamus [27]. In the present study we undertook retrospective review of maternity records to retrieve birthweight and gestational age data, and investigated the relationship between low birthweight and preterm birth and brain structure on MRI during adolescence using qualitative and quantitative MRI analysis methods.

## Methods

### Subjects

One hundred and thirty-seven young people aged 13–22 years receiving additional learning support at their school or college of education were included in this study. The recruitment of these participants has been described elsewhere [24]: in brief, they were recruited via their schools and colleges as part of a larger longitudinal research programme examining the mental health needs of individuals with special education needs for cognitive reasons. Inclusion criteria were the receipt of additional learning support, and age between 13 and 22 years. Exclusion criteria were a history of brain trauma, Down syndrome or other syndromal disorders, major sensory impairments, absence of speech or major cerebral palsy. The intelligence quotient (IQ) of all participants was assessed using the WISC-R [28] for participants under 16 years of age, and the WAIS-R [29] for those over 16 years of age. All participants and their parents or legal guardians provided written informed consent, and ethical permission for the study was received from the Multi-Centre Research Ethics Committee for Scotland.

### Acquisition of obstetric data

Additional written informed consent was sought from all participants and their mothers in order to examine their obstetric records, held centrally by the Information and Statistics Division of the National Health Service of Scotland. Where consent was provided and records were available, data concerning birthweight and gestational age were extracted. Low birthweight was defined as a birthweight of less than 2500 g and preterm birth was defined as a gestational age of less than 37 weeks.

### Image acquisition

All participants were scanned on the same 1.5 T GE MRI scanner (GE Healthcare, Milwaukee, WI) with a 3D inversion-recovery prepared T1-weighted coronal gradient echo sequence, yielding 128 contiguous 1.7 mm coronal slices of 256 × 192 voxels (acquisition parameters: TR/TE/TI/NEX 8.1/3.3/600/1, flip angle 15°, field of view 220 mm). All scans were visually inspected blind to clinical and obstetric data prior to inclusion within the analysis. Scans from an additional 19 participants were excluded from this study: in eighteen cases this was due to insufficient image quality for voxel-based morphometric (VBM) analysis, primarily because of magnetic interference from dental braces and movement artefact, and in one case this was due to massive porencephaly observed in the scan which was incompatible with VBM analysis. The 137 included subjects did not differ from the 19 excluded subjects in terms of gender (χ^2 ^= 1.122, degrees of freedom (df) = 1, *p *= 0.289), age (*F *= 0.397, *p *= 0.530) or IQ (*F *= 0.081, *p *= 0.777).

### Qualitative MRI assessment

All MRI scans were assessed by a neuroradiologist (RJG) experienced in paediatric neuroradiology who was blind to clinical and obstetric details, according to a standardised checklist of qualitative anomalies of brain structure [25]. The checklist comprises 36 separate anomalies of ventricles, other cerebrospinal fluid (CSF) spaces, grey and white matter features as well as other developmental anomalies. Items were defined as normal, moderately abnormal or markedly abnormal (scoring 0, 1 or 2, respectively) as described elsewhere [25]. A total abnormality score for each scan was generated by adding together the scores (0, 1 or 2) for all 36 checklist items. Use of this checklist is associated with a high degree of interobserver and intraobserver agreement [25]. MRI scans were inspected using the multi-planar rendering software, MRIcro [30], and by direct visual inspection of films on a conventional light box.

### Voxel-based morphometry

Image processing was performed using the Statistical Parametric Mapping package (SPM99; The Wellcome Department of Imaging Neuroscience, University College London), running in Matlab version 6.5.1 (The Math Works, Natick, MA). VBM analysis was performed using an adaptation of the optimised methodology proposed by Good et al [31], whereby we used non-linear warping of extracted brains to a study specific template to recover the remapping non-linear warps necessary for scan normalisation, as previously described [27]. To compensate for potential normalisation residuals in the statistical analysis, the normalised total brain volume was taken as a covariate in the analysis of VBM results. All scans were segmented in normalised space using study specific *a priori *tissue maps created specifically for this cohort [31,32]. The normalised grey matter images were smoothed using a 12 mm full width at half maximum (FWHM) Gaussian kernel [33].

### Statistical analysis

Qualitative anomaly scores from subjects with and without a history of low birthweight and prematurity were compared using the two-tailed Mann-Whitney *U *test.

VBM correlation analyses were conducted to examine the linear associations between grey matter density (GMD) and the degree of obstetric adversity (in terms of birthweight and gestational age). Owing to the non-linear nature of the effects of these parameters (the effect of increasing birthweight is different below, within and above the normal birthweight range, and a comparable non-linearity exists for gestational age) obstetric data was transformed from absolute values to figures representing the degree of adversity, as follows: (i) the 'degree of low birthweight' was taken as zero where the birthweight was greater than or equal to 2500 g, or as the difference from 2500 g where the birthweight was below this level; and (ii) the 'degree of prematurity' was taken as zero where the gestational age was 37 weeks or more, or as the difference from 37 weeks where the gestational age was less than this.

All analyses were performed using a multiple regression model in SPM99, and were adjusted for age, gender and normalised total brain volume by including them as covariates. *T*-contrast results were generated as statistical parametric maps with a threshold set at an uncorrected significance level of *T *= 3.2. Voxelwise correction for multiple comparisons was performed using Gaussian random fields theory within SPM99 [33]. Maximum voxel results with *p *< 0.05 (corrected for multiple comparisons) were considered significant, and coordinates of significant voxels were converted from Montreal Neurological Institute (MNI) space to Talairach and Tournoux [34] space using the Matlab script mni2tal.m [35]. Where significant correlations were found, we extracted GMD values for these loci, conducted linear regression analyses in SPSS 14.0 (SPSS for Windows, Rel. 14.0.0. 2005. Chicago: SPSS Inc.), and examined plots of GMD against birthweight and gestational age.

## Results

### Subject characteristics

A total of 137 scans from 85 male and 52 female participants, with mean age 15.9 (SD = 1.6, range 13.1–22.4) and mean IQ 73.0 (SD = 16.5, range 40–131) were included in this study. Obstetric data was available for 98 participants: these participants did not differ from those in whom obstetric data was unavailable in term of gender (χ^2 ^= 0.006, df = 1, *p *= 0.939), age (*F *= 0.059, *p *= 0.809) or IQ (*F *= 1.193, *p *= 0.277).

Birthweight data was available for 90 subjects, of whom 12 were recorded as having low birthweight (< 2500 g), with a mean birthweight of 1730 g (SD = 559.4 g, range 880–2480 g) as compared with a mean of 3399 g (SD = 510.4 g, range 2520–4900 g) among those participants with normal birthweight.

Gestational age data was available for 95 subjects, of whom 13 were recorded as being born prematurely (gestational age < 37 weeks), with a mean gestational age of 33.2 weeks (SD = 3.5 weeks, range 25–36 weeks) as compared with a mean gestational age of 40.4 weeks (SD = 2.2 weeks, range 37–50 weeks) among the remaining participants.

### Qualitative analysis

Low birthweight was associated with significantly higher scores for enlarged subarachnoid cisterns (*U *= 294.0, *Z *= -3.119, *p *= 0.002). Prematurity was associated with significantly higher scores for white matter high intensity of myelination delay (*U *= 492.0, *Z *= -2.512, *p *= 0.012), arachnoid cysts (*U *= 439.0, *Z *= -2.248, *p *= 0.025), enlarged subarachnoid cisterns (*U *= 406.0, *Z *= -2.071, *p *= 0.038) and thinning of the corpus callosum (*U *= 408.5, *Z *= -1.982, *p *= 0.048). No significant associations were detected between these obstetric parameters and the total qualitative abnormality score.

### VBM analysis: correlates of low birthweight (n = 90)

**Figure 1:**
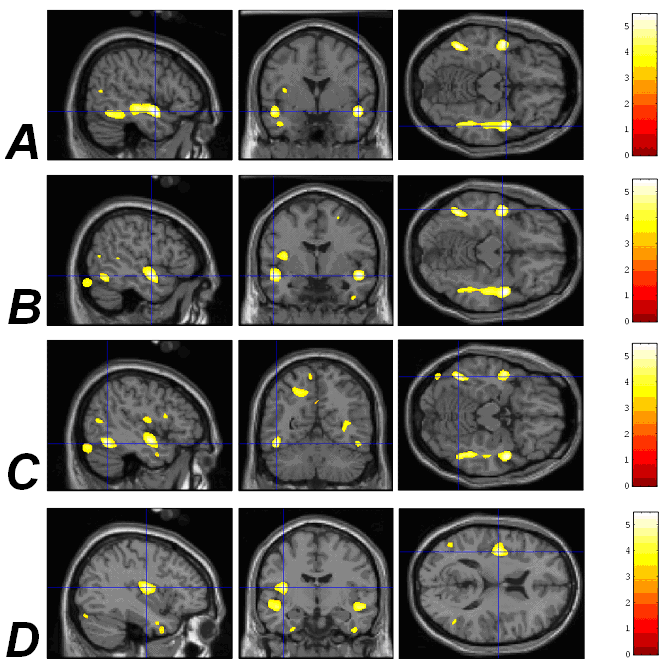
**Correlations between increasingly low birthweight and reduced GMD in different areas of the brain**. (A) The right STG (*p*_corrected _= 0.009). (B) The left STG (*p*_corrected _= 0.031). (C) The left ITG (*p*_corrected _= 0.040). (D) The left insula (*p*_corrected _= 0.049). The coloured bar represents the *Z *statistic for plotted results. Results are plotted onto the SPM99 canonical T1 image for the purpose of illustration.

Significant negative correlations between GMD and the degree of low birthweight were detected in the right superior temporal gyrus (STG; *p*_corrected _= 0.009), left STG (*p*_corrected _= 0.031), left inferior temporal gyrus (ITG; *p*_corrected _= 0.040) and left insula (*p*_corrected _= 0.049); see Figure 1 and Table 1.

**Table 1:** VBM correlations between obstetric adversity and grey matter density. Stereotactic coordinates are quoted within standard Talairach and Tournoux space [34].

**Region**	***x***	***y***	***z***	**Cluster size (voxels)**	***p*-value (maximal voxel)**	**Figures**
**Degree of low birth-weight (*n *= 90), *df *= 85, uncorrected *t *= 3.2**						
						
						
***GMD associations***						
*Positive correlations with GMD*						
None detected						
*Negative correlations with GMD*						
Right STG	50	-1	-12	6131	0.009	1(A), 3(A)
Left STG	-50	-4	-11	2640	0.031	1(B), 3(B)
Left ITG	-46	-55	-11	1497	0.040	1(C), 3(C)
Left insula	-38	-9	12	2098	0.049	1(D), 3(D)
						
**Degree of prematurity (*n *= 95), df = 90, uncorrected *t *= 3.2**						
						
***GMD associations***						
*Positive correlations with GMD*						
None detected						
*Negative correlations with GMD*						
Left STG	-50	-4	-10	2416	0.007	2a, 4a
Right IFG	45	7	28	873	0.009	2b, 4b
Right STG	51	-17	-7	2216	0.026	2c, 4c
Left cerebellar hemisphere	-20	-86	-23	3189	0.036	2d, 4d

### VBM analysis: correlates of prematurity (n = 95)

**Figure 2:**
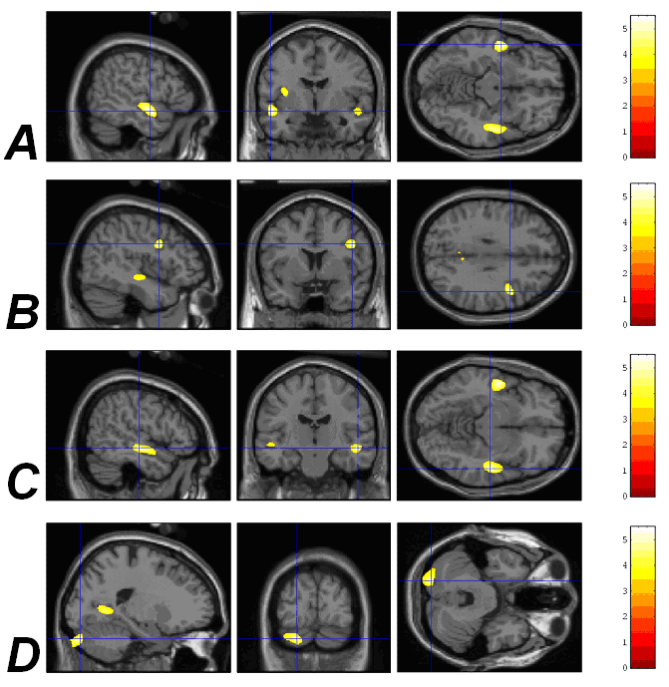
**Correlations between increasing prematurity of birth and reduced GMD in different areas of the brain**. (A) The left STG (*p*_corrected _= 0.007). (B) The right IFG (*p*_corrected _= 0.009). (C) The right STG (*p*_corrected _= 0.026). (D) The left cerebellar hemisphere (*p*_corrected _= 0.036). The coloured bar represents the *Z *statistic for plotted results. Results are plotted onto the SPM99 canonical T1 image for the purpose of illustration.

Significant negative correlations between GMD and the degree of prematurity were detected in the left STG (*p*_corrected _= 0.007), right inferior frontal gyrus (*p*_corrected _= 0.009), right STG (*p*_corrected _= 0.026) and the posterior lobe of the left cerebellar hemisphere (*p*_corrected _= 0.036); see Figure 2 and Table 1.

### Correlations of extracted GMD with birthweight and gestational age

In the case of participants with low birthweight, extracted GMD data from the loci of all significant VBM results demonstrated significant correlations with birthweight for the right STG (*r *= 0.704, *p *= 0.011), the left STG (*r *= 0.742, *p *= 0.006) and the left insula (*r *= 0.716, *p *= 0.009), but not the left ITG (*r *= 0.434, *p *= 0.159). No significant correlations were found to exist between birthweight and GMD data for subjects with birthweight of at least 2500 g.

In the case of participants with preterm birth, extracted GMD data from the loci of all significant VBM results demonstrated significant correlations with gestational age for the left STG (*r *= 0.738, *p *= 0.004), the right IFG (*r *= 0.681, *p *= 0.010), the right STG (*r *= 0.793, *p *= 0.001) and the left cerebellar hemisphere (*r *= 0.564, *p *= 0.045). No significant correlations were found to exist between gestational age and GMD data for subjects with gestational age of at least 37 weeks, although the associations between gestational age and extracted GMD from the right STG and right IFG demonstrated trends towards significance (*r *= 0.204, *p *= 0.066) and (*r *= 0.184, *p *= 0.097), respectively.

**Figure 3:**
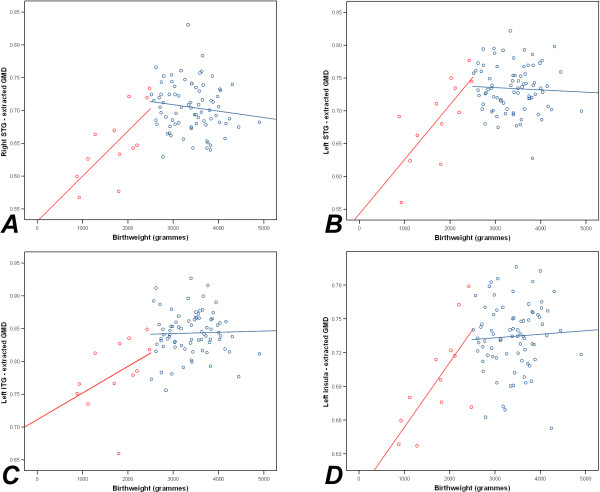
**Scatterplots of birthweight against extracted GMD**. (A) The right STG. (B) The left STG. (C) The left ITG. (D) The left insula. Data from participants with low birthweight (< 2500 g) is shown in red and data from participants with birthweight of at least 2500 g is shown in blue.

**Figure 4:**
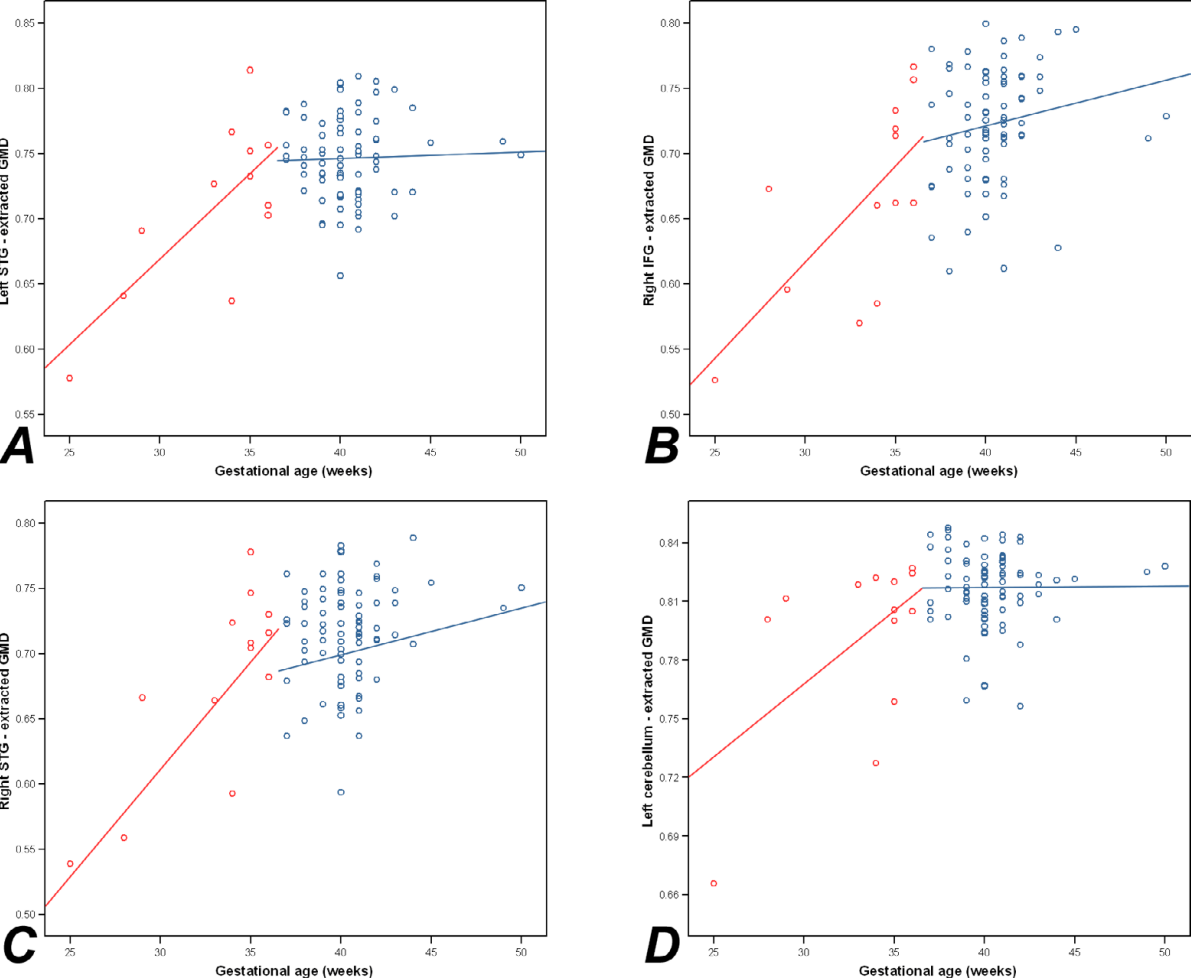
**Scatterplots of gestational age against extracted GMD**. (A) The left STG. (B) The right IFG. (C) The right STG. (D) The left cerebellar hemisphere. Data from participants with preterm birth (< 37 weeks) is shown in red and data from participants with gestational age of 37 weeks or more is shown in blue.

These correlations are presented in Figures 3 and 4, and support the validity of existing thresholds for low birthweight (< 2500 g) and preterm birth (< 37 weeks) against reduced GMD as an outcome measure of adversity. The trends to significant relationships between gestational age and GMD in the right STG and right IFG of non-preterm subjects suggest that there may be a mildly advantageous effect associated with gestational ages beyond 37 weeks. Whilst interesting, this trend requires replication within further and larger samples.

### Comparison with control group (73 versus 72)

It is therefore clear that in this sample recruited from the educational system both low birthweight and preterm birth are associated with an excess of demonstrable structural brain anomalies. The question of the extent to which these obstetric adversities could account for the differences demonstrated between the group with special educational needs as a whole and the controls is obviously raised and this was addressed by comparing (i) a group of 73 participants, comprising the 137 participants investigated within this study, with the exclusion of those with a history of low birthweight or preterm birth and those for whom no birthweight or gestational age data was available; and (ii) the same control group (*n *= 72) as described in our previous report [26], recruited as the cognitively unimpaired siblings, friends and associates of the subjects, and comprising 34 males and 38 females with mean age 16.7 years (SD = 2.1) and mean IQ 101.3 (SD = 15.9).

The group of subjects with no history of low birthweight or preterm birth had significantly higher scores than controls for thinning of the corpus callosum (*U *= 2268.0, *Z *= -2.622, *p *= 0.009), loss of white matter (*U *= 2375.5, *Z *= -2.388, *p *= 0.017), abnormal shape of the lateral ventricles (*U *= 2183.0, *Z *= -2.302, *p *= 0.021) and blunting of the lateral angles of the frontal horns of the lateral ventricles (*U *= 2155.5, *Z *= -2.134, *p *= 0.033); (two-tailed Mann-Whitney U test applied to the data).

## Discussion

Although obstetric data were not universally available, we were able to retrieve birthweight and gestational age data for about 70% of participants and subjects for whom such data were and were not available did not differ on a range of baseline characteristics. One of the most notable findings to emerge from these analyses was the low prevalence of a history of low birthweight and preterm birth within this cohort. Despite the large size of this cohort (we were able to retrieve birthweight and gestational age data for 90 and 95 participants, respectively), only 12 (13.3%) and 13 (13.7%) cases, respectively, had a history of low birthweight and preterm birth. Although this represents an approximately two-fold increase as compared with previously reported prevalences of these conditions within developed world populations as a whole, of 7% (see [4]) and 6% (see [5]), respectively, it indicates that for the overwhelming majority of individuals within the special needs population, aetiological factors other than low birthweight and preterm birth must be considered for the cognitive impairments.

The association between these obstetric adversities and increased rates of occurrence of corpus callosal thinning and arachnoid cysts is consistent with previous qualitative MRI analyses demonstrating similar findings [13,14]. Low birthweight and preterm birth were both associated with enlarged subarachnoid cisterns. Although this anomaly has been reported to be more prevalent among patients with schizophrenia as compared with controls [36], it is generally considered as a benign finding in infants and children [37,38]. No association was found to exist between these obstetric parameters and the total abnormality score, indicating firstly that the non-low-birthweight/preterm participants also had a relatively high rate of structural abnormalities (reflecting the fact that all subjects were in receipt of additional learning support), and also suggesting that the anomalies reported here occur as specific structural associates of low birthweight and prematurity rather than as part of a generalised increase in the overall degree of abnormality of brain structure. This finding is supported by the fact that, following the exclusion of those subjects with a history of low birthweight or preterm birth (and those in whom lack of data meant we were unable to rule out such a history), comparison with a previously defined cognitively unimpaired control group [26] demonstrated increased rates of occurrence of several structural anomalies, including corpus callosal thinning, loss of white matter and lateral ventricular anomalies, providing further evidence for significant structural abnormalities within the non-low-birthweight/preterm subjects with educational difficulties resulting from cognitive impairment.

Our quantitative analyses demonstrated a pattern of abnormalities of brain structure, predominantly within temporal lobe areas, associated with low birthweight and preterm birth. Children born preterm or with low birthweight are at risk of significant cognitive impairment into childhood [6,39]. Given the critical role of temporal lobe structures in language, memory and learning, and the demonstration of abnormalities of structure [8,12,22] and function [40,41] within these brain areas in preterm birth and low birthweight, it is evident that disrupted cortical development within the temporal lobes may be important in the pathogenesis of the cognitive difficulties associated with these peri-natal risk factors.

We also identified a correlation between gestational age and GMD within the left cerebellar hemisphere of individuals born preterm. Previous volumetric studies reported reduced cerebellar volume in adolescents born very preterm [18,19], with significant associations being identified between cerebellar size and cognitive function. Given the traditional association between cerebellar dysfunction and motor in-coordination, these findings may explain the well-documented association between preterm birth and later coordination disorders [42-44].

To our knowledge, this is the first study to investigate the prevalence and neuroanatomical correlates of a history of low birthweight and preterm birth in a clinically well sample of individuals within the special educational needs system. In contrast with previous studies, this sample was not selected in order to identify individuals with a history of obstetric adversities, but instead was recruited via an educational route (in terms of being in receipt of additional learning support at school) and retrospective review of these obstetric parameters was subsequently conducted. This sampling approach lends an important strength to this study, in that the sample of cases is free from sources of bias associated with the use of selected clinic samples, is representative of a large population of individuals within our educational system and lends new insight into previously unaddressed issues affecting this population.

## Conclusion

A recent US Institute of Medicine report estimated that preterm birth in the US cost society US$26.6 billion in 2005, with US$1.1 billion representing the cost of provision of special educational services, and stated the need for outcome studies extending into adolescence [45]. Our findings indicate that, although a two-fold increased prevalence of a history of low birthweight and preterm birth exists within the special educational needs population, other aetiological factors must be considered for the overwhelming majority of cases. Neuroanatomical findings within this sample are concordant with those previously reported within cognitively unimpaired preterm birth/low birthweight cohorts, comprising qualitative anomalies of brain structure and grey matter deficits, particularly of the temporal lobes and cerebellum, that persist into adolescence.

## Competing interests

The author(s) declare that they have no competing interests.

## Authors' contributions

MDS designed the protocol for qualitative analysis, analysed the study data and wrote the first draft of the manuscript. TWJM oversaw the quantitative image analysis methodology. RJG undertook the qualitative assessments of MRI data. AMM supervised the extraction, by JEDS, of data from obstetric records, and assisted in refining statistical analyses. DGCO participated in the recruitment and clinical interviews of participants. SML supervised imaging aspects of the study. ECJ conducted clinical interviews of participants, critically revised the manuscript, obtained programme grant funding for and had overall responsibility for the project and provided overall leadership. All authors participated in meetings to review findings and revise the manuscript.

## Pre-publication history

The pre-publication history for this paper can be accessed here:



## References

[B1] American Association on Mental Retardation (2002). Mental retardation: definition, classification, and systems of supports.

[B2] Roeleveld N, Zielhuis GA, Gabreels F (1997). The prevalence of mental retardation: a critical review of recent literature. Dev Med Child Neurol.

[B3] ISD Scotland (2006). SNS Annual Summary Statistics 2006. http://www.isdscotland.org/isd/4320.html.

[B4] Macfarlane A, Mugford M, Henderson J, Furtado A, Stevens J, Dunn A (2000). Birth Counts: Statistics of Pregnancy and Childbirth.

[B5] Tucker J, McGuire W (2004). Epidemiology of preterm birth. Br Med J.

[B6] Bhutta AT, Cleves MA, Casey PH, Cradock MM, Anand KJ (2002). Cognitive and behavioral outcomes of school-aged children who were born preterm: a meta-analysis. JAMA.

[B7] Linnet KM, Wisborg K, Agerbo E, Secher NJ, Thomsen PH, Henriksen TB (2006). Gestational age, birth weight, and the risk of hyperkinetic disorder. Arch Dis Child.

[B8] Isaacs EB, Edmonds CJ, Chong WK, Lucas A, Morley R, Gadian DG (2004). Brain morphometry and IQ measurements in preterm children. Brain.

[B9] Peterson BS, Vohr B, Staib LH, Cannistraci CJ, Dolberg A, Schneider KC, Katz KH, Westerveld M, Sparrow S, Anderson AW, Duncan CC, Makuch RW, Gore JC, Ment LR (2000). Regional brain volume abnormalities and long-term cognitive outcome in preterm infants. JAMA.

[B10] Indredavik MS, Skranes JS, Vik T, Heyerdahl S, Romundstad P, Myhr GE, Brubakk AM (2005). Low-birth-weight adolescents: psychiatric symptoms and cerebral MRI abnormalities. Pediatr Neurol.

[B11] Skranes JS, Martinussen M, Smevik O, Myhr G, Indredavik M, Vik T, Brubakk AM (2005). Cerebral MRI findings in very-low-birth-weight and small-for-gestational-age children at 15 years of age. Pediatr Radiol.

[B12] Martinussen M, Fischl B, Larsson HB, Skranes J, Kulseng S, Vangberg TR, Vik T, Brubakk AM, Haraldseth O, Dale AM (2005). Cerebral cortex thickness in 15-year-old adolescents with low birth weight measured by an automated MRI-based method. Brain.

[B13] Stewart AL, Rifkin L, Amess PN, Kirkbride V, Townsend JP, Miller DH, Lewis SW, Kingsley DP, Moseley IF, Foster O, Murray RM (1999). Brain structure and neurocognitive and behavioural function in adolescents who were born very preterm. Lancet.

[B14] Peterson BS (2003). Brain imaging studies of the anatomical and functional consequences of preterm birth for human brain development. Ann N Y Acad Sci.

[B15] Nosarti C, Allin MP, Frangou S, Rifkin L, Murray RM (2005). Hyperactivity in adolescents born very preterm is associated with decreased caudate volume. Biol Psychiatry.

[B16] Gimenez M, Junque C, Narberhaus A, Caldu X, Salgado-Pineda P, Bargallo N, Segarra D, Botet F (2004). Hippocampal gray matter reduction associates with memory deficits in adolescents with history of prematurity. Neuroimage.

[B17] Abernethy LJ, Cooke RW, Foulder-Hughes L (2004). Caudate and hippocampal volumes, intelligence, and motor impairment in 7-year-old children who were born preterm. Pediatr Res.

[B18] Allin MP, Salaria S, Nosarti C, Wyatt J, Rifkin L, Murray RM (2005). Vermis and lateral lobes of the cerebellum in adolescents born very preterm. Neuroreport.

[B19] Allin M, Matsumoto H, Santhouse AM, Nosarti C, AlAsady MH, Stewart AL, Rifkin L, Murray RM (2001). Cognitive and motor function and the size of the cerebellum in adolescents born very pre-term. Brain.

[B20] Peterson BS, Anderson AW, Ehrenkranz R, Staib LH, Tageldin M, Colson E, Gore JC, Duncan CC, Makuch R, Ment LR (2003). Regional brain volumes and their later neurodevelopmental correlates in term and preterm infants. Pediatrics.

[B21] Nosarti C, Rushe TM, Woodruff PW, Stewart AL, Rifkin L, Murray RM (2004). Corpus callosum size and very preterm birth: relationship to neuropsychological outcome. Brain.

[B22] Kesler SR, Vohr B, Schneider KC, Katz KH, Makuch RW, Reiss AL, Ment LR (2006). Increased temporal lobe gyrification in preterm children. Neuropsychologia.

[B23] Kesler SR, Ment LR, Vohr B, Pajot SK, Schneider KC, Katz KH, Ebbitt TB, Duncan CC, Makuch RW, Reiss AL (2004). Volumetric analysis of regional cerebral development in preterm children. Pediatr Neurol.

[B24] Johnstone EC, Owens DGC, Hoare P, Gaur S, Spencer MD, Harris J, Stanfield A, Moffat V, Brearley N, Miller P, Lawrie SM, Muir WJ (2007). Schizotypal cognitions as a predictor of psychopathology in adolescents with mild intellectual impairment. Br J Psychiatry.

[B25] Spencer MD, Gibson RJ, Moorhead TWJ, Keston PM, Hoare P, Best JJ, Lawrie SM, Johnstone EC (2005). Qualitative assessment of brain anomalies in adolescents with mental retardation. AJNR Am J Neuroradiol.

[B26] Spencer MD, Moorhead TWJ, Lymer GKS, Job DE, Muir WJ, Hoare P, Owens DGC, Lawrie SM, Johnstone EC (2006). Structural correlates of intellectual impairment and autistic features in adolescents. Neuroimage.

[B27] Spencer MD, Moorhead TWJ, McIntosh AM, Stanfield AC, Muir WJ, Hoare P, Owens DGC, Lawrie SM, Johnstone EC (2007). Grey matter correlates of early psychotic symptoms in adolescents at enhanced risk of psychosis: a voxel-based study. Neuroimage.

[B28] Wechsler D (1992). Wechsler Intelligence Scale for Children (WISC-III-R).

[B29] Wechsler D (1981). Wechsler Adult Intelligence Scale.

[B30] Rorden C, Brett M (2000). Stereotaxic display of brain lesions. Behav Neurol.

[B31] Good CD, Johnsrude IS, Ashburner J, Henson RN, Friston KJ, Frackowiak RS (2001). A voxel-based morphometric study of ageing in 465 normal adult human brains. Neuroimage.

[B32] Moorhead TW, Job DE, Whalley HC, Sanderson TL, Johnstone EC, Lawrie SM (2004). Voxel-based morphometry of comorbid schizophrenia and learning disability: analyses in normalized and native spaces using parametric and nonparametric statistical methods. Neuroimage.

[B33] Ashburner J, Friston KJ (2000). Voxel-based morphometry – the methods. Neuroimage.

[B34] Talairach J, Tournoux P (1988). Co-planar Stereotaxic Atlas of the Human Brain.

[B35] The MNI Brain and the Talairach Atlas. http://www.mrc-cbu.cam.ac.uk/Imaging/Common/mnispace.shtml.

[B36] Bersani G, Quartini A, Piperopoulos O, Iannitelli A, Paolemili M, Pucci D, Di Biasi C, Gualdi G, Pancheri P (2006). Brain abnormalities in schizophrenia. A qualitative comparative study of schizophrenic patients and control individuals assessed by magnetic resonance imaging. J Neuroradiol.

[B37] Barkovich AJ, Barkovich AJ (1999). Hydrocephalus. Pediatric Neuroimaging.

[B38] Nickel RE, Gallenstein JS (1987). Developmental prognosis for infants with benign enlargement of the subarachnoid spaces. Dev Med Child Neurol.

[B39] Anderson P, Doyle LW (2003). Neurobehavioral outcomes of school-age children born extremely low birth weight or very preterm in the 1990s. JAMA.

[B40] Peterson BS, Vohr B, Kane MJ, Whalen DH, Schneider KC, Katz KH, Zhang H, Duncan CC, Makuch R, Gore JC, Ment LR (2002). A functional magnetic resonance imaging study of language processing and its cognitive correlates in prematurely born children. Pediatrics.

[B41] Ment LR, Peterson BS, Vohr B, Allan W, Schneider KC, Lacadie C, Katz KH, Maller-Kesselman J, Pugh K, Duncan CC, Makuch RW, Constable RT (2006). Cortical recruitment patterns in children born prematurely compared with control subjects during a passive listening functional magnetic resonance imaging task. J Pediatr.

[B42] Davis NM, Ford GW, Anderson PJ, Doyle LW (2007). Developmental coordination disorder at 8 years of age in a regional cohort of extremely-low-birthweight or very preterm infants. Dev Med Child Neurol.

[B43] Jongmans MJ, Mercuri E, Dubowitz LMS, Henderson SE (1998). Perceptual-motor difficulties and their concomitants in six-year-old children born prematurely. Hum Movement Sci.

[B44] Holsti L, Grunau RV, Whitfield MF (2002). Developmental coordination disorder in extremely low birth weight children at nine years. J Dev Behav Pediatr.

[B45] Behrman RE, Butler AS (2007). Preterm Birth: Causes, Consequences, and Prevention.

